# Assessment of fluid removal using ultrasound, bioimpedance and anthropometry in pediatric dialysis: a pilot study

**DOI:** 10.1186/s12882-022-03012-1

**Published:** 2023-01-05

**Authors:** Abdulla M. Ehlayel, Oluwatimilehin Okunowo, Mohini Dutt, Kathryn Howarth, Babette S. Zemel, Laura Poznick, Xenia Morgan, Michelle R. Denburg, Lawrence Copelovitch, Susan J. Back, Hansel J. Otero, Erum A. Hartung

**Affiliations:** 1 Division of Nephrology, Children’s Hospital of New Orleans, 200 Henry Clay Ave, New Orleans, LA 70118 USA; 2 Data Science & Biostatistics Unit, Department of Biomedical and Health Informatics, Children’s Hospital of Philadelphia, Philadelphia, USA; 3 Division of Nephrology, Children’s Hospital of Philadelphia, Philadelphia, USA; 4 Division of Gastroenterology, Hepatology and Nutrition, Children’s Hospital of Philadelphia, Philadelphia, USA; 5 Perelman School of Medicine, University of Pennsylvania, Philadelphia, USA; 6 Department of Radiology, Children’s Hospital of Philadelphia, Philadelphia, USA

**Keywords:** Fluid overload, Pediatric hemodialysis, Ultrasound, Anthropometry, Multi-frequency bioimpedance, Isotope dilution

## Abstract

**Background:**

Fluid overload is associated with morbidity and mortality in children receiving dialysis. Accurate clinical assessment is difficult, and using deuterium oxide (D_2_O) to measure total body water (TBW) is impractical. We investigated the use of ultrasound (US), bioimpedance spectroscopy (BIS), and anthropometry to assess fluid removal in children receiving maintenance hemodialysis (HD).

**Methods:**

Participants completed US, BIS, and anthropometry immediately before and 1–2 h after HD for up to five sessions. US measured inferior vena cava (IVC) diameter, lung B-lines, muscle elastography, and dermal thickness. BIS measured the volume of extracellular (ECF) and intracellular (ICF) fluid. Anthropometry included mid-upper arm, calf and ankle circumferences, and triceps skinfold thickness. D_2_O was performed once pre-HD. We assessed the change in study measures pre- versus post-HD, and the correlation of change in study measures with percent change in body weight (%∆BW). We also assessed the agreement between TBW measured by BIS and D_2_O.

**Results:**

Eight participants aged 3.4–18.5 years were enrolled. Comparison of pre- and post-HD measures showed significant decrease in IVC diameters, lung B-lines, dermal thickness, BIS %ECF, mid-upper arm circumference, ankle, and calf circumference. Repeated measures correlation showed significant relationships between %∆BW and changes in BIS ECF (*r*_rm_ =0.51, 95% CI 0.04, 0.80) and calf circumference (*r*_rm_=0.80, 95% CI 0.51, 0.92). BIS TBW correlated with D_2_O TBW but overestimated TBW by 2.2 L (95% LOA, -4.75 to 0.42).

**Conclusion:**

BIS and calf circumference may be helpful to assess changes in fluid status in children receiving maintenance HD. IVC diameter, lung B-lines and dermal thickness are potential candidates for future studies.

**Supplementary Information:**

The online version contains supplementary material available at 10.1186/s12882-022-03012-1.

## Background

Assessment of fluid overload poses a significant challenge in patients with end stage kidney disease (ESKD). This challenge is greater in growing children where it can be difficult to differentiate fluid overload from normal growth and weight gain. Accurate assessment of volume status is important. While fluid overload is associated with increased cardiovascular morbidity and mortality [1], excessive fluid removal can lead to patient discomfort (e.g. dizziness, cramping), accelerated loss of residual kidney function, and myocardial stunning [2]. Assessment of fluid status mainly relies on the estimated dry weight (EDW), a clinically derived estimation of “normal” weight that has significant limitations. Isotope dilution is considered the gold standard for measuring total body water (TBW), but it is too costly and time consuming to be of pragmatic use in clinical practice [3]. Safe, non-invasive, rapid, cost-efficient methods are needed for accurate assessment of volume status in pediatric dialysis patients to guide fluid removal.

Several ultrasound (US) methods have been studied to measure volume status in patients receiving dialysis or with other conditions. Measurements of inferior vena cava (IVC) diameter have been used to assess intravascular volume in critically ill patients and in dialysis [4], and may predict tolerability of fluid removal [5, 6]. On lung US, comet-like artifacts known as B-lines originate from the pleural line and correlate with extravascular lung volume [7], US elastography, which assesses tissue “stiffness,” has been used to measure cutaneous stiffness in various pathologies including lymphedema and systemic sclerosis [8] but has not previously been evaluated in the dialysis population. Given the high content of water in muscles cells [9], muscle elastography may be able to detect changes in muscle stiffness with fluid removal. High-frequency US allows accurate measurement of dermal thickness and has been used to assess and quantify edema in chronic venous disease and subcutaneous edema due to peripheral intravenous catheters [10, 11].

Another non-invasive method used to measure volume status is bioimpedance spectroscopy (BIS), which estimates body water by emitting a small electric current and measuring the resistance to that current as it passes through the body. This allows measurement of intracellular fluid (ICF), extracellular fluid (ECF), and TBW [12]. BIS can determine fluid status, guide fluid removal to achieve EDW, and predict mortality in patients with ESKD [13–15]. While dialysis leads to a decrease in ECF, the effect on ICF varies and may be associated with intradialytic hypotension [16].

Changes in volume status also result in alterations in body size, which may be detected using standardized bedside anthropometric measures such as mid-upper arm circumference (MUAC), calf and ankle circumference, and triceps skinfold thickness. Although anthropometric measures have been studied to assess nutritional status in patients receiving dialysis [17–19], little is known about the relationship between these measures and volume status.

The overall objective of our pilot study was to assess whether changes in fluid status in children on hemodialysis (HD) can be detected using non-invasive methods including US, BIS, and anthropometry. US assessments included IVC diameter, lung B-lines, muscle elastography and dermal thickness. BIS measurements included ECF and ICF. Anthropometric measurements included MUAC, calf and ankle circumference, and triceps skinfold thickness. To evaluate accuracy of BIS compared to “gold standard” isotope dilution using deuterium oxide (D_2_O), we measured TBW using both BIS & D_2_O pre-HD.

We hypothesized that each of the US, BIS, and anthropometric measures would change following HD, and that these changes would correlate with the proportion of body fluid removed during HD. Given that fluid is directly removed from the extracellular compartment with HD, we also hypothesized that changes in US and anthropometric measures would correlate with changes in BIS ECF with HD. In addition, we hypothesized that pre-HD TBW measured by BIS would correlate with the gold standard D_2_O TBW.

## Methods

### Study design

This prospective, observational cohort study was approved by the Children’s Hospital of Philadelphia (CHOP) Institutional Review Board (IRB #18-015039). Written informed consent was obtained from all legal guardians or participants 18 years or older, and child assent was obtained as appropriate.

Participants completed US, BIS, and anthropometry immediately before HD and 1–2 h after HD to allow vascular refilling. Pre- and post-HD study measures were repeated for each participant on up to 5 separate HD sessions to capture varying levels of volume status.

On a separate visit, TBW was measured using D_2_O before HD only. US, BIS, and anthropometry were also obtained at this visit before dialysis. Measures were not repeated after HD due to length of the visit.

### Setting and participants

Participants receiving maintenance HD were recruited from the CHOP outpatient HD unit between January 2019 and February 2020. Inclusion criteria were age ≥ 1 year old and on HD for > 1 month at the time of enrollment, to allow time for stabilization on HD. The age of 1 year was chosen due to physical limitations of applying BIS electrodes. Patients with conditions affecting IVC, such as heart failure and IVC thrombus, or skin lesions interfering with BIS probe placement were excluded.

### Study procedures

US images were obtained during a 30 min session and anthropometry/BIS were done in a separate 30 min session. Sessions were completed consecutively. The order of sessions (US vs. anthropometry/BIS) was completed based on scheduling availability. US images were obtained in the supine position and anthropometric measurements in the standing position. For BIS, participants were asked to lay flat on a table for a minimum of 5 min before obtaining measurements. A summary of study measurements is presented in Supplementary Table S1.

#### Physical exam and clinical data

A physical exam was completed by investigators (AE, XM or EH) prior to a scheduled HD session on the day of the study visit. The exam included auscultation of the lungs for crackles, and evaluation for periorbital, sacral, and lower limb edema. Investigators obtained pre-HD blood pressure (BP) measurement by auscultation. Post-HD BP readings were retrieved from the medical record and obtained immediately post-HD by oscillometry, as per HD unit protocol. Clinical data was collected from participants’ medical records at the time of the visit and between study visits, including vital signs, medications, lab results and hospitalizations. Dialysis treatment settings and EDW were determined by the clinical team. Patients were allowed to eat and drink per HD unit policy. Residual kidney function was not measured.

#### Ultrasound

US assessment of IVC, lung, and muscle elastography were obtained using GE Logiq-E9 or E10 (GE Healthcare, Chicago, IL). A convex probe was used for the IVC measurements and linear probe for the lung and muscle elastography. Dermal thickness was obtained using Vevo 2100 (FUJIFILM VisualSonics, Toronto, CA) with ultra-high frequency probe. Images were obtained in 2-dimensional B-mode. IVC diameter was measured once to the right of the xiphoid. IVC measurements included minimum (IVC_min_) diameter, maximum (IVC_max_) diameter, and IVC collapsibility index (IVCCI). IVC diameter changes during the respiratory cycle, with the largest diameter noted during expiration and “collapsing” to the smallest diameter during inspiration. IVCCI reflects the proportion of change in the diameter during inspiration, with less collapse and therefore a lower IVCCI noted with fluid overload.

Lung US images were obtained in the intercostal space at 9 positions in each lung, for a total of 18 lung windows: upper, mid and lower lung at the mid-clavicular, anterior axillary, and mid-axillary lines. The total number of B-lines from all windows was used for analysis.

**Fig. 1:**
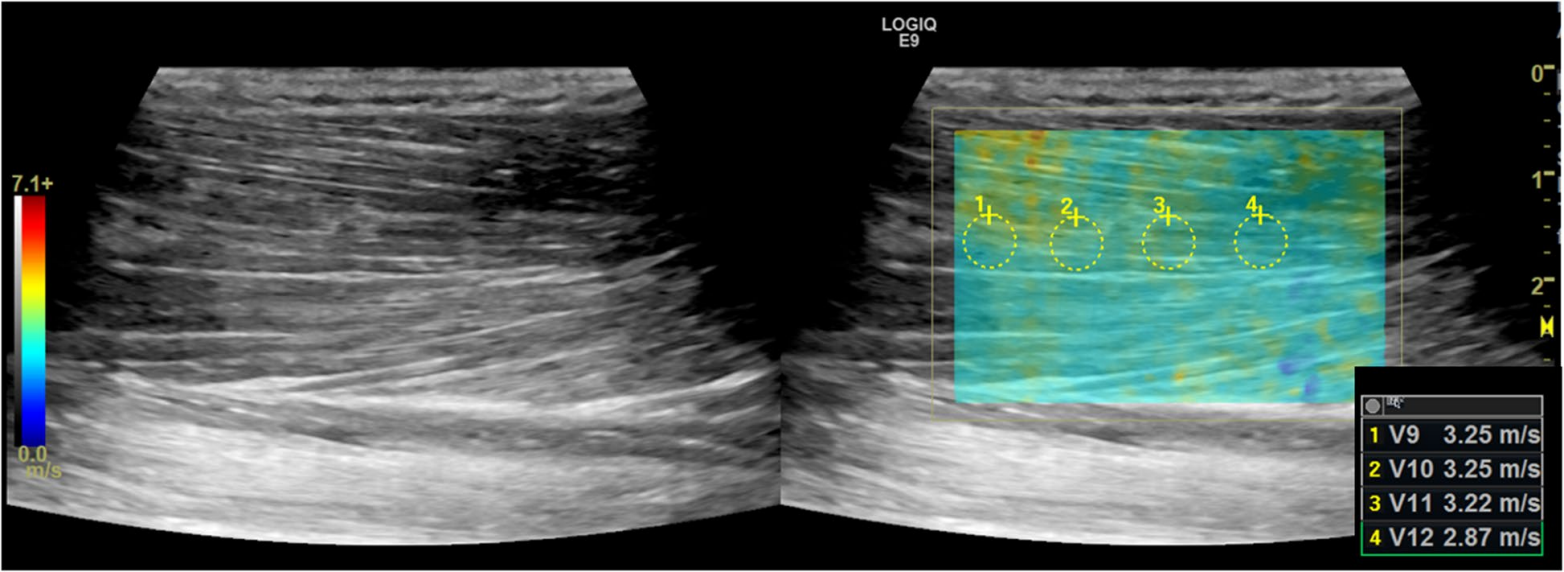
Muscle elastography ultrasound image. Grayscale ultrasound image (left) and shear wave elastography map (right) of the gastrocnemius. The elastography map (blue box) represents the interrogated tissue and the small circles are drawn regions of interest, each representing a velocity measurement (shear wave velocity), which can be directly translated into muscle stiffness

**Fig. 2:**
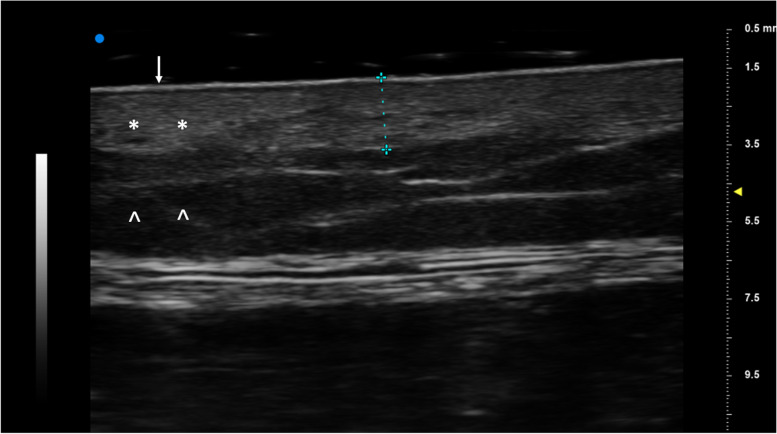
High frequency skin ultrasound image. High frequency ultrasound image of the pretibial soft tissues show the echogenic epidermis (arrow), heterogeneous and less echogenic dermis (*) and the hypoechoic hypodermis (^). (+ - - - +) Represents study measurement

Muscle elastography was obtained on the lateral aspect of the right leg. The median value from at least 8 shear wave velocity regions of interest was used for analysis (Fig. 1). Dermal thickness (mm) was measured on the medial aspect of the right lower leg on the surface of the tibial bone (Fig. 2). The average of 3 measurements was used for analysis.

All US images were obtained by qualified pediatric sonographers and interpreted by two pediatric radiologists who were blinded to the patient’s clinical status, including weight and volume status. Interpretation of imaging results was conducted independently by the radiologists.

#### Bioimpedance spectroscopy

Whole body BIS was obtained using Xitron Hydra 4200 after participants were in a supine position for at least 5 min. Two electrodes were placed on the dorsum of the hand and two on the dorsum of the foot, as per manufacturer recommendations [20]. Three consecutive measurements were obtained. None of the participants had a fistula on the side of measurement.

Estimates of the extracellular fluid (ECF) and intracellular fluid (ICF) were generated by the BIS program and used for analysis. The Hydra 4200 estimates ECF and ICF using equations formulated from Hanai mixture theory [20]. BIS TBW was calculated as a sum of ECF and ICF. The average of 3 readings was used for analysis.

#### Anthropometry

Anthropometric measurements of MUAC, calf and ankle circumference, and triceps skinfold thickness were obtained by research anthropometrists using standardized methods as described in the Anthropometric Standardization Reference Manual [21]. Limb circumferences were measured with a non-stretchable fiberglass tape (± 0.1 cm; Weigh and Measures LLC, MD) without interfering clothing. Triceps skinfold thickness was measured using skinfold calipers (Holtain, UK). Triplicate measurements were taken from the arm without the HD vascular access and the average of 3 measurements was used for analysis.

Standing weight and height measurements were also obtained. Weight (± 0.1 kg, Scaletronix, NY) was obtained before and after HD with participants wearing minimal clothing. The average of three measurements was used. Height was obtained pre-HD using a stadiometer (± 0.1 cm, Holtain, UK) and the average of 2 measurements was used in the analysis. Percent change in body weight (%∆BW) was calculated as follows:

$$\mathit\%\Delta BW=\left(\frac{predialysis\;weight-postdialysis\;weight}{predialysis\;weight}\right)\times100$$

#### Isotope dilution

For the isotope dilution study visit, a baseline blood sample was drawn and then participants were given 0.15 g/kg of D_2_O (99.9% enrichment, Cambridge Isotope Laboratories) 4 h before their scheduled HD session. The D_2_O water was administered orally from a syringe under supervision of study personnel. An accurate weight of administered D_2_O was calculated from the difference in weight of the syringe before and after administration of D_2_O using a precision scale (± 0.0001 g, Mettler-Toledo scale model AG104, OH). Blood samples for D_2_O level measurement were drawn at baseline and 4 h after administration, prior to initiation of HD. Participants were asked to void prior to administration of D_2_O and the first blood draw. Urine voided during study period was measured by study investigators. Blood samples were stored at -80° C until study completion and analyzed in batch by cavity ring-down spectroscopy (Metabolic solutions, NH). Samples were injected 10 times and the results of the last 3 measurements were averaged to mitigate between sample memory effects [22], with a precision of 2 parts per thousand. D_2_O TBW was calculated at the equilibration time point from isotope dilution space assuming TBW = N_D_/1.041 to correct for isotopic fractionation [23]. TBW percentage (%TBW) was calculated as follows:

$$\%TBW=\left(\frac{TBW}{body\;weight}\right)\times100$$

### Statistical methods

Continuous data are reported as median (interquartile range), mean (standard deviation [SD]), and categorical data as counts (percentages). We used a mixed-effects model to estimate the change in study measures before and after the initiation of dialysis. From this model, we reported the estimates before and after baseline. The intraclass correlation coefficient (ICC) which measures the degree of correlation within clusters was also reported. The ICC ranges from 0 to 1. An ICC of 0 means all the observations are independent of one another. A nonzero ICC suggests variability and that the observations are not independent [24].

Using the repeated measures correlation function “rmcorr()”, we assessed the relationship between within-person changes pre- and post-HD in US measures, BIS, anthropometry, and BP with %∆BW. Repeated measures correlation was also used to assess the relationship between within-person changes pre- and post-HD in US measures and anthropometry with BIS ECF. Repeated measures correlation is a statistical technique for determining within-individual association for paired measures assessed at two or more time points for multiple individuals [25]. The rmcorr coefficient (*r*_rm_) is bound by -1 to 1 and represents the strength of the linear association between two variables [25]. Observations missing pre- or post-HD values were not included in the primary analysis. In sensitivity analyses, missing values were substituted with multiple imputation procedures using the “mice()” function from the R multivariate imputation by chained equation (MICE) package.

Bland-Altman method was used to assess agreement between BIS and D_2_O measures of TBW. This analysis determines the mean differences (or bias) between tests as a measure of accuracy, where small bias indicates high accuracy. The 95% Limit of Agreement (LOA) was defined by ± 1.96 SD of the bias. A narrow 95% LOA means high precision of measurement [26].

The level of significance was set at *p*-value < 0.05. Data management and analyses were conducted with SAS version 9.4 (SAS Institute Inc., Cary, NC, USA) and R software version 4.0.5 (R Core Team, 2021).

## Results

### Demographics

Nine children undergoing maintenance HD were enrolled in the study with one participant withdrawing consent prior to completing any study measures. Patient demographics and clinical characteristics are presented in Table 1. Eight subjects were included in the analysis (6 males), median age 17.0 years (range 3.4–18.5). Median duration on HD was 3.4 months (range 1.4–75.2). Five out of 8 participants (62.5%) were on antihypertensive medications. No participants had diabetes. A total of 29 pre/post visits (median 4.5 visits per participant) and 6 isotope study visits were completed (one participant received a kidney transplant and another participant refused D_2_O study). Urine output measured during D_2_O study period was negligible in amount. Physical exam findings of edema were absent in most study visits. Six visits were missing some study measures. Details of missing data and study visits are presented in Supplementary Table S2. The technical error of measurement was assessed with root mean square of error and % coefficient of variation. Results in Supplementary Table S3.

**Table 1:** Patient demographics and clinical characteristics

	1	2	3	4	5	6	7	8	Summary (median or %)
Age (years)	3.4	9.7	10.4	16.8	17.2	17.4	17.5	18.5	17.0
Gender	F	M	M	M	F	M	M	M	M 75%
Race	W	W	AA	AA	AA	AA	AA	AA	AA 75%
Cause of ESKD	Aplasia	MCD	Obstructive uropathy	FSGS	FSGS	FSGS	Dysplasia	Obstructive uropathy	
Duration on HD (months)	21.9	4.1	75.2	5.8	2.3	1.4	2.7	2.3	3.4
Vascular access	Catheter	Catheter	Catheter	Catheter	Catheter	Fistula	Catheter	Fistula	Catheter 75%
**Dialysis settings (avg per session)**									
* Duration (hr)*	3.9	4.2	4.2	3.7	4.2	3.6	3.3	4.1	4
* Prescribed Qb (ml/min)*	86	200	150	350	350	350	400	440	350
* UF volume (L)*	0.2	1.0	1.2	3.8	2.7	2.1	2.4	3.2	2.25
Number of paired pre/post visits	2	5	2	5	1	5	5	4	4.5
Number of anti-HTN medications	0	2	2	0	2	3	0	3	2.5
HTN medication class	-	CCB α1-blocker	CCB α1-blocker	-	CCB α1-blocker	ARB α1-blocker Β-blocker	-	CCB α1-blocker ACEI	anti-HTN medication 62.5%
Growth hormone	Yes	No	Yes	No	No	No	No	No	Yes 25%
Completed isotope visit	Yes	Yes	No	Yes	No	Yes	Yes	Yes	Yes 75%

*AA* African American, *ACEI* Angiotensin converting enzyme inhibitor, *ARB* Angiotensin receptor blocker, *avg* average, *CCB* Calcium channel blocker, *ESKD* End-stage kidney disease, *F* Female, *FSGS* Focal segmental glomerulosclerosis, *HD* Hemodialysis, *HTN* Hypertension, *M* Male, *MCD* Minimal change disease, *min* minutes, *Qb* blood flow, *UF* Ultrafiltration, *W* White

### Change in Study measures pre & post HD

Average pre-HD weight for the cohort was 54.3 kg, decreasing to 52.4 kg post HD. Mean systolic BP decreased from 125.8 to 113.7 mmHg and mean diastolic BP decreased from 74.7 to 68.2 mmHg. Change in study measures pre- to post- HD are presented in Table 2. On US, mean IVC measurements showed a significant decrease in IVC_min_ and IVC_max_ diameters (*p* < 0.05). The mean total number of lung B-lines decreased from 12.5 to 9.4 (*p* = < 0.0001). Average dermal thickness decreased by 0.12 mm (*p* = 0.02). Intraclass coefficient showed moderate to high agreement in most study measures between radiologists.

Mean BIS ECF volume decreased by 1.4% (*p* = < 0.0001) and mean BIS ICF increased by 1.7% (*p* = < 0.0001). Mean MUAC decreased by 0.5 cm and calf measurements by 0.6 cm (*p* = < 0.0001 for both). Mean ankle circumference decreased by 0.3 cm (*p* = 0.01).

**Table 2:** Change in study measures pre-post hemodialysis

	Average pre dialysis (SD)	Average post dialysis (SD)	Change in average study measure [average post-pre]	*P*-value	ICC
**Clinical Parameters**					
Weight (kg)	54.3 (11.3)	52.4 (11.3)	-1.9	**< 0.0001**	0.99
SBP (mmHg)	125.8 (6.9)	113.7 (6.94)	-12.1	**< 0.0001**	0.97
DBP (mmHg)	74.7 (5.0)	68.2 (5.0)	-6.5	**0.003**	0.96
**Ultrasound**					
IVC_min_ (cm)	1.2 (0.2)	1.0 (0.2)	-0.16	0.04	0.37
IVC_max_ (cm)	1.5 (0.2)	1.3 (0.2)	-0.24	0.002	0.47
IVCCI	0.2 (0.03)	0.2 (0.03)	-0.02	0.5	0
Lung B-lines (n)	12.5 (5.2)	9.4 (5.2)	-3.2	< 0.0001	0.99
Muscle Elastography (m/s)	2.6 (0.2)	2.7 (0.2)	0.05	0.75	0.14
Dermal Thickness (mm)	1.4 (0.15)	1.3 (0.15)	-0.12	0.02	0.49
**Bioimpedance**					
BIS %ECF [%ECF = ECF/Wt*100]	25.0(0.9)	23.6 (0.9)	-1.4	**< 0.0001**	0.76
BIS ECF (L)	13.2 (2.3)	12.0 (2.3)	-1.3	**< 0.0001**	0.97
BIS %ICF [%ICF = ICF/Wt*100]	30.5 (1.7)	34.3 (1.7)	3.8	**< 0.0001**	0.88
BIS ICF (L)	16.9 (3.9)	18.6 (3.9)	1.7	**< 0.0001**	0.99
**Anthropometry**					
Triceps Skinfold Thickness (mm)	10.7 (1.7)	10.4 (1.7)	-0.3	0.253	0.96
MUAC (cm)	25.6 (2.7)	25.1 (2.7)	-0.5	**< 0.0001**	0.99
Calf Circumference (cm)	32.2 (2.8)	31.6 (2.8)	-0.6	**< 0.0001**	0.99
Ankle Circumference (cm)	19.6 (1.5)	19.3 (1.5)	-0.3	**0.01**	0.97

*BIS* Bioimpedance, *cm* centimeter, *DBP* Diastolic blood pressure, *ECF* Extracellular fluid, *ICC* Intraclass correlation coefficient, *IVC* Inferior vena cava, *IVCCI* IVC collapsibility index, *mm* millimeter, *ICF* Intracellular fluid, *kg* kilograms, *L* Liters, *m/s* meter/second, *max* maximum, *min* minimum, *mmHg* millimeters of mercury, *MUAC* Mid-upper arm circumference, *n* number, *SD* Standard deviation, *SBP* Systolic blood pressure, *Wt* Weight

Average of study measures were compared before and after hemodialysis. For participants with more than one visit, the average of the individual participant’s study measure was included in the analysis. ICC ranges from 0 to 1, where nonzero value suggests variability and that the observations are not independent.

### Correlation of change in study measures with %∆BW

%∆BW showed a statistically significant and moderate correlation with change in BIS ECF (*r*_rm_= 0.51, 95% CI 0.04 to 0.80, *p* = 0.03) and a strong correlation with change in calf circumference (*r*_rm_= 0.8, 95%CI 0.51 to 0.92, *p* < 0.0001). Correlation of change in remaining study measures with %∆BW did not meet the threshold for statistical significance (Table 3). In sensitivity analysis with missing values imputed, no significant correlations were found between change in study measures and %∆BW (Supplementary Table S4).

**Table 3:** Correlation of change in study measures and clinical parameters with %∆BW

Measurement	*r*_rm_ (95% CI)	*P*-value
**Ultrasound**		
Change in IVC_min_	-0.04 (-0.51, 0.45)	0.9
Change in IVC_max_	0.16 (-0.35, 0.60)	0.5
Change in IVCCI	-0.04 (-0.51, 0.45)	0.9
Change in Lung B-lines	-0.41 (-0.74, 0.10)	0.1
Change in Muscle Elastography	0.03 (-0.48, 0.52)	0.9
Change in Dermal Thickness	0.26 (-0.29, 0.68)	0.3
**Bioimpedance**		
Change in ICF	-0.11 (-0.56, 0.39)	0.6
Change in ECF	**0.51 (0.04, 0.80)**	**0.03**
**Anthropometry**		
Change in Triceps Skinfold Thickness	0.29 (-0.22, 0.67)	0.2
Change in MUAC	0.27 (-0.25, 0.66)	0.3
Change in Calf Circumference	**0.80 (0.51, 0.92)**	**< 0.0001**
Change in Ankle Circumference	0.08 (-0.41, 0.54)	0.7
**Clinical Parameters**		
Change in SBP	0.15 (-0.36, 0.59)	0.6
Change in DBP	-0.09 (-0.55, 0.41)	0.7

*%∆BW* Percent change in body weight, *DBP* Diastolic blood pressure, *ECF* Extracellular fluid, *IVC* Inferior vena cava, *IVCCI* IVC collapsibility index, *ICF* Intracellular fluid, *max* maximum, *min* minimum, *SBP* Systolic blood pressure

### Correlation of change in study measures with BIS ECF

Assessment of correlation of change in study measures with change in BIS ECF showed no statistically significant relationships in primary analysis (Table 4) and sensitivity analysis (Supplementary Table S5).

**Table 4:** Correlation of change in study measures with change in ECF measured by BIS

Measurement	*r*_rm_ (95% CI)	*P*-value
**Ultrasound**		
Change in IVC_min_	-0.34 (-0.70, 0.17)	0.2
Change in IVC_max_	-0.19 (-0.61, 0.32)	0.4
Change in IVCCI	0.24 (-0.27, 0.65)	0.3
Change in Lung B-lines	-0.28 (-0.67, 0.24)	0.3
Change in Muscle Elastography	-0.004 (-0.50, 0.49)	0.9
Change in Dermal Thickness	0.02 (-0.50, 0.52)	0.9
**Anthropometric Measurements**		
Change in Triceps Skinfold Thickness	-0.06 (-0.52, 0.44)	0.8
Change in MUAC	0.05 (-0.44, 0.52)	0.8
Change in Calf Circumference	0.29 (-0.22, 0.68)	0.2
Change in Ankle Circumference	-0.02 (-0.50, 0.47)	0.9

*BIS* Bioimpedance, *ECF* Extracellular fluid, *IVC* Inferior vena cava, *IVCCI* IVC collapsibility index, *ICF* Intracellular fluid, *max* maximum, *min* minimum, *MUAC* Mid-upper arm circumference

### BIS TBW compared to D_2_O TBW

**Fig. 3:**
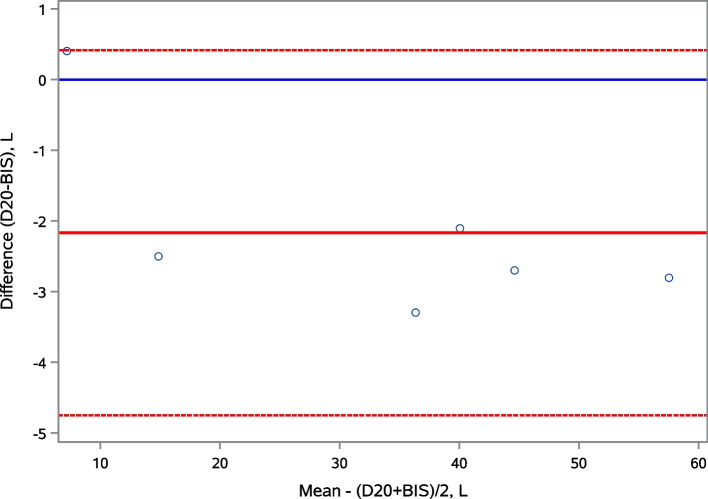
Bland-Altman plots showing agreement between TBW assessed by D_2_O and by BIS. Solid red line: mean of the difference. Dashed lines: 95% limits of agreement (LOA). BIS: bioimpedance spectroscopy, D_2_O: deuterium oxide, L: liters, TBW: total body water

Mean BIS TBW was 34.5 L, compared to the gold standard mean D_2_O TBW of 32.4 L, showing that BIS TBW significantly overestimated TBW compared to D_2_O by 2.2 +/- 1.3 L (95% LOA, -4.75 to 0.42) (Fig. 3).

## Discussion

In this pilot study, we evaluated the utility of various US, BIS, and anthropometric measures to assess fluid removal in children receiving maintenance HD. Multiple measures showed significant changes after HD, but BIS ECF and calf circumference were the only study measures to show correlation with %∆BW. The change in study measures before and after HD supports the potential utility of these measures in assessing volume status in children on dialysis and a possible role in guiding fluid removal.

In our study, average IVC_min_ and IVC_max_ decreased after HD but showed no correlation with %∆BW in individual-level analysis. The change in IVC diameter following HD is consistent with prior studies in children [27, 28]. A study in 16 children on maintenance HD evaluating hydration status with echocardiography and BIS found a significant decrease in IVC_min_ and IVC_max_ following HD when compared to pre-HD [27]. This change did not correlate with overhydration on BIS. IVCCI reflects proportion of change in IVC diameter and inversely correlates with central venous pressure in children with heart disease [29] and has been proposed for fluid assessment in patients with ESKD [30]. In a study by Haciomeroglu et al. in children on dialysis and healthy controls, IVCCI significantly increased after HD and approached that of controls, but did not correlate with ultrafiltration [28]. Our study did not show a significant change in IVCCI after HD, nor a correlation with %∆BW. This may relate to limitations of the study including the small sample size. The lack of correlation between fluid removal and IVC diameter in our study and previous studies may be due to the presence of excess fluid mainly in the extravascular space in HD patients, which may limit the utility of IVC in assessing total excess volume. Challenges of IVC measurements in younger children include coordination of IVC measurements with the respiratory cycle and operator-dependent nature of this study.

B-lines on lung US reflect extravascular lung water and are typically absent in healthy individuals. Adult and pediatric studies have shown a decrease in the number of lung B-lines with dialysis [7, 31–33] and correlation in the number of lung B-lines with changes in body fluid status [32, 34]. In our study, the average number of B-lines decreased post-HD, consistent with prior studies [7, 34]. However, we did not observe a statistically significant relationship between change in B-lines and %∆BW, likely due to the small number of participants in our study. Although we did not detect a relationship between B-lines and fluid removal, some literature suggests B-lines may be used to objectively quantify fluid overload. In a study using 28 lung windows, Noble et al. reported that the number of B-lines decreased by 2.7 for every 500 mL of volume removed [32]. Due to its non-invasive nature and relative ease and portability, lung US has potential to be feasible in clinical dialysis settings. Typical limitations of lung US such as the inability to differentiate between B-lines due to volume overload from those due to interstitial pulmonary fibrosis, heart failure, or ARDS [35] are less prevalent in outpatient pediatric dialysis population, which may make this technique more suitable for clinical adoption.

We investigated two novel US methods to assess fluid status in children on HD: muscle elastography to measure muscle stiffness and high-frequency US to measure dermal thickness. In our subjects, muscle stiffness did not change after HD and did not correlate with %∆BW. This may be due to the lack of change in muscle stiffness with changes in water content, or perhaps due to predominance of fluid removal from the intravascular compartment during HD and insufficient wait time to allow equilibration with the extravascular compartment. Better understanding of the relationship between muscle elastography and fluid status is needed before using this method for fluid assessment in HD. We found that dermal thickness decreased after HD and showed a positive but not statistically significant correlation with fluid removal. In prior studies, US was able to detect changes in dermal thickness in healthy adult volunteers following fluid infusion and in adult patients following HD [36, 37]. However, there is no reference data on normal pediatric dermal thickness on US. Additional studies will be needed to establish reference data and quantify the change in response to fluid removal to make dermal high-frequency US clinically meaningful. With increased availability of portable US, dermal US may be a potential candidate for non-invasive fluid assessment since images can be obtained quickly with minimal training.

Anthropometric measurements are simple, inexpensive methods to assess body composition at the bedside. MUAC and calf circumference are used to determine fat free mass, and triceps skinfold thickness measures subcutaneous fat [19]. Given that excess fluid is found in the dermis and may be reflected in these measurements, we explored the potential role of these methods in fluid assessment. In our study, MUAC and calf circumference decreased with HD, as expected. Change in calf circumference showed a strong correlation with %∆BW suggesting a significant change in the “volume” of calf muscle. Although the absolute change is relatively small, this strong correlation suggests the calf may be a good candidate to assess change in fluid status on dialysis, potentially when combined with segmental BIS of the calf. Surprisingly, change in calf circumference did not show a correlation with BIS ECF. We expected a correlation given the large water content in muscles [9]. The lack of correlation may be due to the limitations of precision of the BIS device, measurement of calf circumference, or the small number of subjects. Ankle circumference showed significant decrease but did not correlate with %∆BW. We found that triceps skinfold thickness, a measure that is typically used to evaluate nutritional status, did not change following HD and was not correlated with %∆BW. This finding suggests that triceps skinfold thickness is not influenced by body fluid status, which provides some reassurance that it may provide a consistent assessment of nutritional status in children on HD without interference from volume overload.

Bioimpedance has been proposed as a rapid, non-invasive method for fluid assessment. However, clinical application remains challenging due to several reasons including lack of manufacturing standards, variability in measurements related to technique, and the inability to use devices interchangeably [34]. In addition, the large discrepency in BIS estimates compared to dilution methods in dialysis patients limits clinical applicability [38, 39]. We used the Xitron Hydra 4200b to assess change in BIS estimates in relation to HD. The preceding generation of this device has been validated in healthy subjects relative to isotope dilution [40, 41]. Despite published concerns regarding the BIS estimates of the device, we used the values provided by the machine to reflect clinical application of the device. As expected, mean BIS %ECF decreased with HD and BIS ECF showed a positive correlation with %∆BW. Although an increase in ICW following HD is unexpected, it has been proposed that the rapid decrease in extracellular BUN may result in delayed osmotic equilibration between ECW and ICW, leading to water movement into the intracellular compartment [42–44]. This observation has also been reported by other investigators using bioimpedance [16]. The change in absolute BIS estimates of TBW (ECF + ICF) were significantly different than average change in weight (Table 2), consistent with findings reported by Milani et al. regarding the precision of BIS in dialysis [38]. The relationship between change in ECF and %∆BW was modest (*r* = 0.51), likely reflecting the limitations of TBW estimates in this population. When compared to the gold standard of D_2_O, BIS TBW showed good correlation but significantly overestimated TBW by 2.2 L. This bias, possibly more prominent in younger children due to higher TBW to body weight ratio [38], is significant considering that this difference may be larger than absolute weight change during a single HD session. Our results are consistent with findings that have found wide LOA when comparing bioimpedance to deuterium [38, 39]. The accuracy of prediction of ECF and ICF using BIS depends on assumed values for tissue resistivity which in turn is affected by several factors, making it difficult to compare data using different devices [45]. In addition, this device has not been validated in children on HD to our knowledge. Bioimpedance estimates seem to be consistent at a population level but not on an individual basis [46]. This suggest that BIS may be useful in monitoring the relative change in fluid rather than the absolute value of estimated volume. Our findings also suggest that the BIS device algorithms to calculate TBW in this device may be less accurate in children on HD compared to D_2_O. Nevertheless, the portable, non-invasive nature of BIS makes this method appealing as a potential candidate for future studies.

Average systolic and diastolic BP decreased after HD, but the decrease in BP did not correlate with %∆BW. This is consistent with prior studies that showed fluid overload contributes to an elevated BP but is not the only factor [47]. Change in BP may be due to other factors including white-coat effect, technique of measurement, and increased renin secretion. In our patients, clinical exam findings of fluid overload were lacking at most study visits and provided limited input in the assessment of fluid status.

Collectively, several study measures showed promising results warranting additional investigation and correlation of use with outcome. Lung US, dermal US, and BIS are non-invasive techniques that can be used at the bedside with a portable device, and anthropometric measures such as calf circumference can be performed without the need for specialized equipment. Potential scenarios could include the use of a combination of these non-invasive techniques to assess “fluid overload score” in patients at regular intervals to help guide clinicians in fluid removal. Other uses could include evaluation for volume overload in patients with acute kidney injury, heart failure, or liver disease. These measures could also be helpful to monitor change of fluid status from baseline in patients at risk of volume overload and could aid decision-making regarding timing of dialysis initiation.

Our pilot study has several strengths and limitations. Strengths include repeated measures in each participant, temporal relationship of study measures to dialysis, rigorous study methodology, and use of D_2_O isotope dilution as a gold standard. Study measures were repeated on different days to capture variations of volume status. Radiologic and anthropometric studies were obtained by trained personnel to ensure consistent and reproducible measurements. Although this may not be feasible in other settings with limited resources, it provided a more accurate assessment of the study measures. The main limitation is the small number of participants in a single center, which limited our power to detect some differences. However, our study provides encouraging data to further explore noninvasive measures such as US, BIS, and anthropometrics to inform clinical decisions regarding fluid removal for children on dialysis.

## Supplementary Information

**Additional file 1: Supplementary Table S1.** Summary of study measurement details and corresponding units. **Supplementary Table S2.** Missing data study visits. **Supplementary Table S3.** Root mean square of error (RMSE) and %coefficient of variation (%CV) for study visits. **Supplementary Table S4.** Sensitivity analysis for correlation of change in study measures and clinical parameters with percent change in body weight (%∆BW). **Supplementary Table S5.** Sensitivity analysis for correlation of change in study measures with change in extracellular fluid measured by bioimpedance spectroscopy (BIS ECF). 

## Data Availability

The datasets used and/or analysed during the current study are available from the corresponding author on reasonable request.

## Abbreviations

%∆BW: Percent change in body weight
BIS: Bioimpedance spectroscopy
CHOP: Children’s Hospital of Philadelphia
D_2_O: Deuterium oxide
ECF: Extracellular fluid
EDW: Estimated dry weight
ESKD: End stage kidney disease
HD: Hemodialysis
ICC: Intraclass correlation coefficient
ICF: Intracellular fluid
IVC: Inferior vena cava
IVCCI: Inferior vena cava collapsibility index
IVC_max_: Inferior vena cava maximum diameter
IVC_min_: Inferior vena cava minimum diameter
LOA: Limits of agreement
MUAC: Mid-upper arm circumference
*r*_rm_: rmcorr coefficient
TBW: Total body water
US: Ultrasound

## References

[1] Onofriescu M, Siriopol D, Voroneanu D, Voroneanu L, Hogas S, Nistor I, Apetrii M (2015). Overhydration, cardiac function and survival in hemodialysis patients. Lionetti V, editor. PLoS One.

[2] Eng CSY, Bhowruth D, Mayes M, Stronach L, Blaauw M, Barber A, et al. Assessing the hydration status of children with chronic kidney disease and on dialysis: a comparison of techniques. Nephrol Dial Transplant. 2017;(March):1–9. Available from: http://academic.oup.com/ndt/article/doi/10.1093/ndt/gfx287/4609370. 10.1093/ndt/gfx287 29136192

[3] Foster BJ, Leonard MB (2004). Measuring nutritional status in children with chronic kidney disease. Am J Clin Nutr.

[4] Kaptein MJ, Kaptein EM (2017). Focused real-time ultrasonography for nephrologists. Int J Nephrol.

[5] Kaptein M, Kaptein J, Oo Z, Kaptein E (2018). Relationship of inferior vena cava collapsibility to ultrafiltration volume achieved in critically ill hemodialysis patients. Int J Nephrol Renovasc Dis.

[6] Krause I, Birk E, Davidovits M, Cleper R, Blieden L, Pinhas L (2001). Inferior vena cava diameter: a useful method for estimation of fluid status in children on haemodialysis. Nephrol Dial Transplant.

[7] Allinovi M, Saleem M, Romagnani P, Nazerian P, Hayes W (2016). Lung ultrasound: a novel technique for detecting fluid overload in children on dialysis. Nephrol Dial Transplant.

[8] DeJong HM, Abbott S, Zelesco M, Kennedy BF, Ziman MR, Wood FM. The validity and reliability of using ultrasound elastography to measure cutaneous stiffness, a systematic review. Int J Burns Trauma. 2017;7(7):124–41. Available from: https://www.ncbi.nlm.nih.gov/pmc/articles/PMC5768929/. PMC5768929 29348976

[9] Forbes RM, Cooper AR, Mitchell HH (1953). The composition of the adult human body as determined by chemical analysis. J Biol Chem.

[10] Yabunaka K, Murayama R, Tanabe H, Takahashi T, Oe M, Oya M (2016). Ultrasonographic classification of subcutaneous edema caused by infusion via peripheral intravenous catheter. J Med Ultrasound.

[11] Volikova AI, Edwards J, Stacey MC, Wallace HJ (2009). High-frequency ultrasound measurement for assessing post-thrombotic syndrome and monitoring compression therapy in chronic venous disease. J Vasc Surg.

[12] Scotland G, Cruickshank M, Jacobsen E, Cooper D, Fraser C, Shimonovich M (2018). Multiple-frequency bioimpedance devices for fluid management in people with chronic kidney disease receiving dialysis: a systematic review and economic evaluation. Health Technol Assess (Rockv).

[13] Yang EM, Park E, Ahn YH, Choi HJ, Kang HG, Cheong H, Il (2017). Measurement of fluid status using bioimpedance methods in korean pediatric patients on hemodialysis. J Korean Med Sci.

[14] Zoccali C, Moissl U, Chazot C, Mallamaci F, Tripepi G, Arkossy O (2017). Chronic fluid overload and mortality in ESRD. J Am Soc Nephrol.

[15] Ponce P, Pham J, Gligoric-Fuerer O, Kreuzberg U. Fluid management in haemodialysis: conventional versus body composition monitoring (BCM) supported management of overhydrated patients. Port J Nephrol Hypertens. 2014;28(3):239–48. Retrieved December 05, 2022, from http://scielo.pt/scielo.php?script=sci_arttext&pid=S0872-01692014000300007&lng=en&tlng=en.

[16] Ismail AH, Gross T, Schlieper G, Walter M, Eitner F, Floege J (2021). Monitoring transcellular fluid shifts during episodes of intradialytic hypotension using bioimpedance spectroscopy. Clin Kidney J.

[17] Dahl H, Warz S-I, Welland NL, Arnesen I, Marti H-P, Dierkes J. Factors associated with nutritional risk in patients receiving haemodialysis assessed by Nutritional Risk Screening 2002 (NRS2002). J Ren Care. 2021. Available from: http://www.ncbi.nlm.nih.gov/pubmed/33977653. 10.1111/jorc.12374 33977653

[18] Peng H, Aoieong C, Tou T, Tsai T, Wu J (2021). Clinical assessment of nutritional status using the modified quantified subjective global assessment and anthropometric and biochemical parameters in patients undergoing hemodialysis in Macao. J Int Med Res.

[19] Chumlea WC. Anthropometric and body composition assessment in dialysis patients. Semin Dial. 17(6):466–70. Available from: http://www.ncbi.nlm.nih.gov/pubmed/15660577. 10.1111/j.0894-0959.2004.17607.x 15660577

[20] Hydra ECF/ICF. (Model 4200) operating manual. Available from: https://vitrek.com/downloads/legacy/xitron/hydramanual.pdf. Cited 2022 Apr 22.

[21] Lohman TG, Roache AF, Martorell R (1988). Anthropometric standardization reference manual.

[22] Penna D, Stenni B, S̊anda M, Wrede S, Bogaard TA, Michelini M (2012). Technical note: evaluation of between-sample memory effects in the analysis of δ2H and δ18O of water samples measured by laser spectroscopes. Hydrol Earth Syst Sci.

[23] Heymsfield SB, Lohman TG, Wang Z, Going SB, editors. Human body composition. Human Kinetics; 2005. Available from: https://www.humankineticslibrary.com/encyclopedia?docid=b-9781492596950.

[24] Park S, Lake ET. Multilevel modeling of a clustered continuous outcome: nurses’ work hours and burnout. Nurs Res. 54(6):406–13. Available from: http://www.ncbi.nlm.nih.gov/pubmed/16317362. 10.1097/00006199-200511000-00007 PMC1540459 16317362

[25] Bakdash JZ, Marusich LR. Repeated measures correlation. Front Psychol. 2017;8. Available from: https://www.frontiersin.org/article/10.3389/fpsyg.2017.00456/full. 10.3389/fpsyg.2017.00456 PMC5383908 28439244

[26] Bland JM, Altman DG (1986). Statistical methods for assessing agreement between two methods of clinical measurement. Lancet.

[27] Torterüe X, Dehoux L, Macher M-A, Niel O, Kwon T, Deschênes G (2017). Fluid status evaluation by inferior vena cava diameter and bioimpedance spectroscopy in pediatric chronic hemodialysis. BMC Nephrol.

[28] Haciomeroglu P, Ozkaya O, Gunal N, Baysal K (2007). Venous collapsibility index changes in children on dialysis. Nephrology (Carlton).

[29] Iwamoto Y, Tamai A, Kohno K, Masutani S, Okada N, Senzaki H (2011). Usefulness of respiratory variation of inferior vena cava diameter for estimation of elevated central venous pressure in children with cardiovascular disease. Circ J.

[30] Kraemer M, Rode C, Wizemann V (2006). Detection limit of methods to assess fluid status changes in dialysis patients. Kidney Int.

[31] Vitturi N, Dugo M, Soattin M, Simoni F, Maresca L, Zagatti R (2014). Lung ultrasound during hemodialysis: the role in the assessment of volume status. Int Urol Nephrol.

[32] Noble VE, Murray AF, Capp R, Sylvia-Reardon MH, Steele DJR, Liteplo A (2009). Ultrasound assessment for extravascular lung water in patients undergoing hemodialysis: Time course for resolution. Chest.

[33] Trezzi M, Torzillo D, Ceriani E, Costantino G, Caruso S, Damavandi PT (2013). Lung ultrasonography for the assessment of rapid extravascular water variation: evidence from hemodialysis patients. Intern Emerg Med.

[34] Allinovi M, Saleem MA, Burgess O, Armstrong C, Hayes W. Finding covert fluid: methods for detecting volume overload in children on dialysis. Pediatr Nephrol. 2016. 10.1007/s00467-016-3431-4 PMC5118410 27282380

[35] Covic A, Siriopol D, Voroneanu L. Use of lung ultrasound for the assessment of volume status in CKD. Am J Kidney Dis. 2017. 10.1053/j.ajkd.2017.10.009. 10.1053/j.ajkd.2017.10.009 29274919

[36] Eisenbeiss C, Welzel J, Eichler W, Klotz AK (2001). Influence of body water distribution on skin thickness: measurements using high-frequency ultrasound. Br J Dermatol.

[37] Brazzelli V, Borroni G, Vignoli GP, Rabbiosi G, Cavagnino A, Berardesca E (1994). Effects of fluid volume changes during hemodialysis on the biophysical parameters of the skin. Dermatology.

[38] Milani GP, Groothoff JW, Vianello FA, Fossali EF, Paglialonga F, Edefonti A (2017). Bioimpedance and fluid status in children and adolescents treated with dialysis. Am J Kidney Dis.

[39] Raimann JG, Zhu F, Wang J, Thijssen S, Kuhlmann MK, Kotanko P (2014). Comparison of fluid volume estimates in chronic hemodialysis patients by bioimpedance, direct isotopic, and dilution methods. Kidney Int..

[40] Patel RV, Matthie JR, Withers PO, Peterson EL, Zarowitz BJ (1994). Estimation of total body and extracellular water using single- and multiple-frequency bioimpedance. Ann Pharmacother.

[41] Moon JR, Tobkin SE, Roberts MD, Dalbo VJ, Kerksick CM, Bemben MG (2008). Total body water estimations in healthy men and women using bioimpedance spectroscopy: a deuterium oxide comparison. Nutr Metab.

[42] Tapolyai MB, Faludi M, Fülöp T, Dossabhoy NR, Szombathelyi A, Berta K (2014). Which fluid space is affected by ultrafiltration during hemodiafiltration?. Hemodial Int.

[43] Singh AT, Mc Causland FR (2017). Osmolality and blood pressure stability during hemodialysis. Semin Dial.

[44] Henrich WL, Woodard TD, Blachley JD, Gomez-Sanchez C, Pettinger W, Cronin RE (1980). Role of osmolality in blood pressure stability after dialysis and ultrafiltration. Kidney Int.

[45] Ward LC, Isenring E, Dyer JM, Kagawa M, Essex T (2015). Resistivity coefficients for body composition analysis using bioimpedance spectroscopy: Effects of body dominance and mixture theory algorithm. Physiol Meas.

[46] Piccoli A (2014). Estimation of fluid volumes in hemodialysis patients: comparing bioimpedance with isotopic and dilution methods. Kidney Int.

[47] Zaloszyc A, Schaefer B, Schaefer F, Krid S, Salomon R, Niaudet P (2013). Hydration measurement by bioimpedance spectroscopy and blood pressure management in children on hemodialysis. Pediatr Nephrol.

