# Supplementary material for: Assessment of fluid removal using ultrasound, bioimpedance and anthropometry in pediatric dialysis: a pilot study
Source: BMC Nephrol. 2023 Jan 5;24:5. doi: 10.1186/s12882-022-03012-1 (PMC9814436; doi:10.1186/s12882-022-03012-1)
Supplement: Supplementary file 1 — Additional file 1: Supplementary Table S1. Summary of study measurement details and corresponding units. Supplementary Table S2. Missing data study visits. Supplementary Table S3. Root mean square of error (RMSE) and %coefficient of variation (%CV) for study visits. Supplementary Table S4. Sensitivity analysis for correlation of change in study measures and clinical parameters with percent change in body weight (%∆BW). Supplementary Table S5. Sensitivity analysis for correlation of change in study measures with change in extracellular fluid measured by bioimpedance spectroscopy (BIS ECF). [file 12882_2022_3012_MOESM1_ESM.pdf]

Supplementary Table S1: Summary of study measurement details and corresponding units

Study Measurement	Measurement Details	Unit of measurement
Ultrasound		
IVC	IVCCI= $(IVC_{max}-IVC_{min})/IVC_{max}$<br><br>$IVC_{min}$= Diameter of IVC during inspiration<br>IVC_{max}= Diameter of IVC during expiration	Diameter, cm
Lung	B-lines per US field	n= total number of B-lines
Muscle	US elastography for muscle stiffness of the anterior leg compartment	Shear wave velocity, m/s
Skin	Ultra-high frequency US to assess pretibial thickness of the dermis	Thickness, mm
Bioimpedance		
ECF	Multi-frequency BIS to estimate ECF	Volume, L
%ECF	(ECF/weight) *100	%
ICF	Multi-frequency BIS to estimate ICF	Volume, L
%ICF	(ECF/weight) *100	%
Anthropometry		
Weight	Standardized measurement	kg
Height	Standardized measurement	cm
Arm, calf and ankle circumference	Standardized measurement of the mid-upper arm, max calf and ankle	Circumference, cm
Triceps skinfold thickness	Standardized measurement	Thickness, mm
**Isotope Dilution**		
Isotope dilution using D_2O	Measurement of blood D_2O concentration 4 hours after administration	Volume, L

cm: centimeter, D_2O: deuterium oxide, L: liters, mm: millimeter, kg: kilogram, m/s: meters/second, IVC: inferior vena cava, IVCCI: IVC collapsibility index, max: maximum, min: minimum, MUAC: mid-upper arm circumference, US: ultrasound; ECF: extracellular fluid; ICF: intracellular fluid

Supplementary Table S2 - Missing data study visits

Participant Number	Missing Data	Reason
2, visit #5	Pre visit ultrasound, All post measures at the same visit	Patient declined
8, visit #1	Pre skin US	Machine malfunction
6, visit #3 & #5	Pre skin US on 2 separate visits	Not documented
6, visit #3	All post measures	Hospital Admission
7, visit #3	All post measures	Scheduling error

Supplementary Table S3- Root mean square of error (RMSE) and %coefficient of variation (%CV) for study visits

	RMSE	%CV
PRE-calf	0.059	0.193
PRE-ankle	0.075	0.382
PRE-arm circumference	0.088	0.332
PRE-triceps	0.349	2.820
PRE-BIS ECF	1.711	14.619
PRE-BIS ICF	2.576	14.652
POST-calf	0.217	0.567
POST-ankle	0.067	0.356
POST-arm circumference	0.053	0.215
POST-triceps	0.402	3.283
POST-BIS ECF	0.060	0.408
POST-BIS ICF	0.184	0.981

Supplementary Table S4- Sensitivity analysis for correlation of change in study measures and clinical parameters with percent change in body weight (%ΔBW)

**Measurement**	**r_{rm} (95% CI)**	**p-value**
**Ultrasound**		
Change in IVC_{min}	-0.03 (-0.47, 0.42)	0.9
Change in IVC_{max}	0.13 (-0.33, 0.54)	0.6
Change in IVCCI	-0.05 (-0.48, 0.40)	0.8
Change in Lung B-lines	-0.27 (-0.63, 0.20)	0.2
Change in Muscle Elastography	-0.002 (-0.44, 0.44)	0.9
Change in Dermal Thickness	0.17 (-0.29, 0.57)	0.4
**Bioimpedance**		
Change in ICF	-0.01 (-0.45, 0.44)	0.9
Change in ECF	0.28 (-0.19, 0.64)	0.2
**Anthropometry**		
Change in Triceps Skinfold Thickness	0.18 (-0.29, 0.58)	0.4
Change in MUAC	0.03 (-0.42, 0.47)	0.9
Change in max Calf Circumference	0.12 (-0.34, 0.53)	0.6
Change in Ankle Circumference	-0.01 (-0.45, 0.43)	0.9
**Clinical Parameters**		
Change in SBP	0.07 (-0.38, 0.50)	0.7
Change in DBP	-0.11 (-0.53, 0.35)	0.6

%ΔBW: percent change in body weight, DBP: diastolic blood pressure, ECF: extracellular fluid, IVC: inferior vena cava, IVCCI: IVC collapsibility index, ICF: intracellular fluid, max: maximum; min: minimum, SBP: systolic blood pressure

Supplementary Table S5– Sensitivity analysis for correlation of change in study measures with change in extracellular fluid measured by bioimpedance spectroscopy (BIS ECF)

**Measurement**	**r_{rm} (95% CI)**	**p-value**
**Ultrasound**		
Change in IVC_{min}	-0.07 (-0.50, 0.38)	0.8
Change in IVC_{max}	-0.01 (-0.45, 0.43)	0.9
Change in IVCCI	0.18 (-0.29, 0.58)	0.4
Change in Lung B-lines	-0.14 (-0.55, 0.32)	0.5
Change in Muscle Elastography	0.04 (-0.41, 0.48)	0.9
Change in Dermal Thickness	0.11 (-0.35, 0.52)	0.6
**Anthropometric Measurements**		
Change in Triceps Skinfold Thickness	-0.18 (-0.58, 0.28)	0.4
Change in MUAC	0.13 (-0.33, 0.54)	0.6
Change in max Calf Circumference	0.22 (-0.25, 0.60)	0.3
Change in Ankle Circumference	0.11 (-0.35, 0.52)	0.6

BIS: bioimpedance, ECF: extracellular fluid, IVC: inferior vena cava, IVCCI: IVC collapsibility index, ICF: intracellular fluid, max: maximum; min: minimum, MUAC: mid-upper arm circumference
